# Pediatric Respiratory Infections ‎After the COVID-19 Pandemic: A Single-Center Experience

**DOI:** 10.7759/cureus.65779

**Published:** 2024-07-30

**Authors:** Walaa A Shahin, Khaled Alamri, Eshraq Omar, Yousef Elmahmoud, Hayam H Ahmed, Faisal Al Enezi, Ghada Alshaman, Abdulrahman Alodayani, Hassan Alahmari

**Affiliations:** 1 Pediatric Pulmonology, Cairo University Pediatric Hospital, Cairo, EGY; 2 Advanced General Pediatrics, Prince Sultan Military Medical City, Riyadh, SAU; 3 Pediatric Infectious Diseases, Prince Sultan Military Medical City, Riyadh, SAU

**Keywords:** respiratory distress, flu, pediatric infections, influenza, rsv, bronchiolitis, covid-19, lower respiratory tract infection, infants, kingdom of saudi arabia (ksa)

## Abstract

Background

Pediatric respiratory infections, mainly bronchiolitis, are a substantial clinical burden. The most common etiology is respiratory syncytial virus (RSV). Other viruses include human rhinovirus, human metapneumovirus, influenza, adenovirus, coronavirus, and parainfluenza viruses.

Objective

We aimed to study the epidemiology and clinical characteristics of children with confirmed viral bronchiolitis and flu after the COVID-19 pandemic season and compare the behavior of each virus.

Methods

This retrospective observation study was done over seven months, from October 2022 to April 2023. All children (0-14) were included in the study if they met the clinical diagnosis of bronchiolitis or flu. Viral etiology is confirmed by PCR, using the respiratory panel available in our center which included the detection of four viruses: COVID-19, RSV, influenza A, and B. Clinical data, lab results, and X-rays were collected and correlated with each viral infection for all admitted patients.

Results

We recruited 237 children with bronchiolitis and flu symptoms from October 2022 to April 2023. The peak of infections (41%) was in November. Seasonal variations for each virus showed distinct patterns across the year. RSV peaked at the beginning of the season, gradually declining after that. In contrast, influenza A and B maintained a relatively consistent presence throughout the season. Meanwhile, COVID-19 reached its peak during March and April. One hundred forty-four (60%) of the patients were under two years of age. RSV was predominant in 150 patients (63.3%). COVID-19 was only detected in 25 patients (10%), whereas influenza A and B were equally isolated in 31 (13%) patients each. Fifty-one children (21%) were initially sick and required pediatric intensive care unit (PICU) admission, with no deaths reported. Notably, COVID-19 had a milder disease course, a shorter length of stay (LOS) in the hospital (two days) and a shorter duration of illness (five days) compared to other viruses. RSV infection was linked to more profound hypoxia and more sick children with more extended hospital stays.

Conclusion

Our study showed that, following the pandemic and the release of lockdown measures, there was another peak of upper respiratory tract infections (URTI) and flu, which was more aggressive, primarily due to other viruses, especially RSV. This resurgence was associated with more severe respiratory symptoms and an increased need for hospitalization. Notably, children with COVID-19 were in better condition compared to those with RSV.

## Introduction

Bronchiolitis typically affects infants and young children with signs of respiratory distress and a lower respiratory tract infection. The diagnosis is based mainly on the clinical symptoms [1]. It is often described as a virus-induced inflammation of small bronchioles and their surrounding tissue. According to different guidelines, the upper age limit varies from six to 12 months, with 12 months preferred by many European countries and up to two years used in the United States [2].

Respiratory syncytial virus (RSV) is the predominant organism responsible for around 70% of bronchiolitis cases worldwide; the prevalence in the Kingdom of Saudi Arabia has a wide variation of 25%-88% [3]. Data have shown that bronchiolitis cannot simply be diagnosed using a certain cut-off age but instead uses viral etiology as the differentiating factor [2].

The most common etiology of bronchiolitis is RSV, with the highest incidence of infection between December and March in North America; however, regional variations occur. Infection with RSV does not lead to long-life immunity, with possible reinfections throughout life. Other viruses that cause bronchiolitis include human rhinovirus, human metapneumovirus, influenza, adenovirus, coronavirus, and parainfluenza viruses [4].

RSV-associated acute lower respiratory infection (ALRI) is a significant cause of hospital admissions in young children, resulting in a major concern for health care services. Globally, nearly 45% of hospitalizations and in-hospital deaths due to RSV-ALRI occur in children during the first six months of life [5].

Early childhood RSV-ALRI has been reported to have an association with the subsequent development of wheeze-associated disorders in childhood [6]. There is international attention on the RSV vaccine, which is focused on the implications of the novel vaccine and its potential long-term effects, especially on subsequent childhood wheezes and asthma development. This interest may lead to the broader application of maternal RSV vaccination and the implementation of future antiviral strategies [5].

Seasonal influenza is an acute respiratory infection caused by viruses circulating worldwide. There are four types of influenza viruses: A, B, C, and D. Influenza A and B viruses circulate and cause seasonal epidemics [7].

Influenza is a significant cause of morbidity and mortality worldwide. It annually infects 5-15% of the global population, resulting in an estimated 250,000-500,000 deaths per year [8]. Pharmacologic interventions are not routinely recommended for the management of immune-competent infants and children with non-severe bronchiolitis who were treated in the office or emergency department because they lack the evidence for their efficacy, increase the cost of care, and may even lead to adverse effects. Randomized trials, systematic reviews, and meta-analyses do not support the benefits of bronchodilators (inhaled or oral), glucocorticoids (inhaled or systemic), or leukotriene inhibitors. Antibiotics are indicated only if there is evidence of a coexisting bacterial infection. This approach is consistent with that of the American Academy of Pediatrics, the National Institute for Care Excellence, and other professional organizations [9-12].

Regarding investigations such as blood gas, complete blood counts, serum electrolytes, and urinalysis or urine culture, they have remained the same in the past decade. They are indicated only in cases of severe respiratory distress or impending failure. Serious bacterial infections associated with bronchiolitis are rare, and universal guidelines (AAP 2014, NICE 2015, CPS Canada, and France 2013) recommend against complete blood counts and cultures unless young infants are evaluated for possible sepsis. Hydration status should be assessed and monitored by clinical examination. Unfortunately, despite the many high-quality international guidelines for diagnosing and managing bronchiolitis, there is a lot of faulty practice among physicians who request unnecessary investigations [1].

We aimed in this study to identify the clinical characteristics of children with confirmed viral bronchiolitis and flu in the season after the COVID-19 pandemic, which will allow us as healthcare providers to know the impact of COVID-19 on the immune response of children after the lockdown and after the pandemic has gone. This knowledge will enable us to evaluate current treatment modalities and improve and modify the services provided to these children and their families, especially during the high season. Additionally, it will help in identifying those who are at greater risk of developing complications.

## Materials and methods

This was a retrospective study aimed at identifying and studying the clinical characteristics of children with confirmed respiratory viral illness who presented to a tertiary hospital in Prince Sultan Military Medical City (PSMMC), Riyadh, Saudi Arabia, for a period of seven months from October 2022 to April 2023. All children (0-14 years old) were included in the study if they met the clinical diagnosis of bronchiolitis or flu. The viral etiology was confirmed by the reverse transcription polymerase chain reaction (RT-PCR) technique, using the respiratory panel available and approved by hospital policy to screen children with URTI in our center, which included the detection of four viruses: COVID-19, RSV, influenza A, and influenza B. Other viruses are to be requested individually by the treating physician. Children were excluded from the study if their nasopharyngeal swap came to be negative or if the required data were missing. Clinical data, lab results, and X-rays were collected for all admitted patients. Data were collected by the authors and plotted in an Excel sheet (Microsoft® Corp., Redmond, WA), followed by statistical analysis using the Statistical Product and Service Solutions (SPSS, version 28; IBM SPSS Statistics for Windows, Armonk, NY) program.

Data were coded and entered using SPSS. Data were summarized using mean, standard deviation, median, minimum, and maximum in quantitative data, as well as frequency (count) and relative frequency (percentage) for categorical data. Comparisons between quantitative variables were done using the non-parametric Kruskal-Wallis and Mann-Whitney tests. The chi-square test was performed to compare categorical data. The exact test was used when the expected frequency was less than 5. P-values less than 0.05 were considered statistically significant.

## Results

Table 1 shows that the total number of recruited patients was 237 children who presented to the ER with flu symptoms and upper or lower respiratory tract infection during the season of October 2022-April 2023, with a peak (41%) in November. Sixty percent of the children were under two years of age. RSV was predominant (63.3%), while COVID-19 and influenza A and B were equally distributed around 10 and 13%, respectively. Of the critically ill patients who required a pediatric intensive care unit (PICU), 21% had a complete recovery, and no deaths were reported.

**Table 1:** General demographic data of the study population (October 2022 to April 2023)‎

Variable		Count N=237	Percent
Month	Oct	35	14.8%
	Nov	99	41.8%
	Dec	34	14.3%
	Jan	22	9.3%
	Feb	12	5.1%
	Mar	10	4.2%
	Apr	25	10.5%
Diagnosis	Influenza A	31	13.1%
	Influenza B	31	13.1%
	COVID-19	25	10.5%
	RSV	150	63.3%
Age in months	0-24	144	61%
	25-60	56	23.5%
	>60	37	15.5%
Children with co-morbidity	Yes	80	33.8%
Children with congenital heart disease (CHD)	Yes	12	5.5%
Admission to PICU	Yes	51	21.5%
Outcome	Improved	202	85.2%
	Revisit to ED	35	14.8
	Death	0	0

Table 2 shows the clinical presentation of the studied population. Nearly 50% of the children who presented to the ED had hypoxia and wheezy chest, for which they received systemic and inhaled steroids with inhaled bronchodilator. Around half of the patients’ chest X-rays showed interstitial infiltration and bronchial thickening. There were 80 (34%) children with underlying co-morbidities, 20 of them (25%) required intensive care and more respiratory support in the PICU. The total number of patients admitted to the PICU was 51, less than half of them (20) had underlying co-morbidities in the form of prematurity, neuromuscular disorder, and Down syndrome. However, all of them recovered.

**Table 2:** Clinical data of the studied population CPAP: continuous positive airway pressure; HFNC: high-flow nasal cannula; PICU: pediatric intensive care unit

Clinical sign	N=237	Percent	
Hypoxia (Sat ≤ 95)	122	51%	
Wheezes	110	46.4%	
Conjunctivitis	6	2.5%	
PICU mode of support (N=51)	51	21.5%	
CPAP	8	3.4%	
HFNC	37	15.6%	
Intubated	6	2.5%	
Co-morbidities in PICU	20	20%	
Systemic steroids	115	48.5%	
Inhaled bronchodilator	160	67.5%	
Inhaled steroids	109	46.0%	
Chest X-ray			
Hyperinflation	29	12.2%	
Atelectasis	78	32.9%	
Interstitial infiltration	99	41.8%	
Bronchial thickening	141	59.5%	
Normal	15	6.3%	

Table 3 shows some laboratory data of the studied population where the average hospital stay was five days and the PICU was three days.

**Table 3:** Some laboratory and numerical data of the studied population

Finding	Minimum	Maximum	Median
Degree of fever on admission °C	36.7	40.2	38
Initial oxygen saturation	68%	99%	95%
Days in PICU	1	30	3
Total leucocytic count TLC	1.46	41.50	10.16
Lymphocytes 10^9^/L	0.27	15.68	3.48
Neutrophils 10^9^/L	0.17	35.83	4.28
CRP mg/L	0.03	332.00	10.48
PCT micg/L	0.00	49.60	0.23
Total Days of illness	2.00	48.00	9.00
Length of hospital stay in days	1	41	5.00

**Figure 1:**
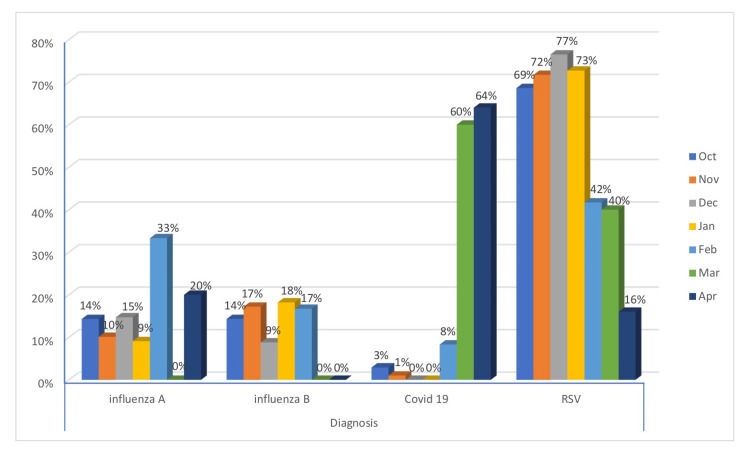
The monthly variation of viral infections (October 2022-April 2023)‎

Seasonal variations for each virus were illustrated in Figure 1, showing distinct patterns across the year. RSV had a peak at the beginning of the season, gradually declining thereafter. In contrast, influenza A and B maintained a relatively consistent presence throughout the entire season. Meanwhile, COVID-19 reached its peak during March and April.

**Figure 2:**
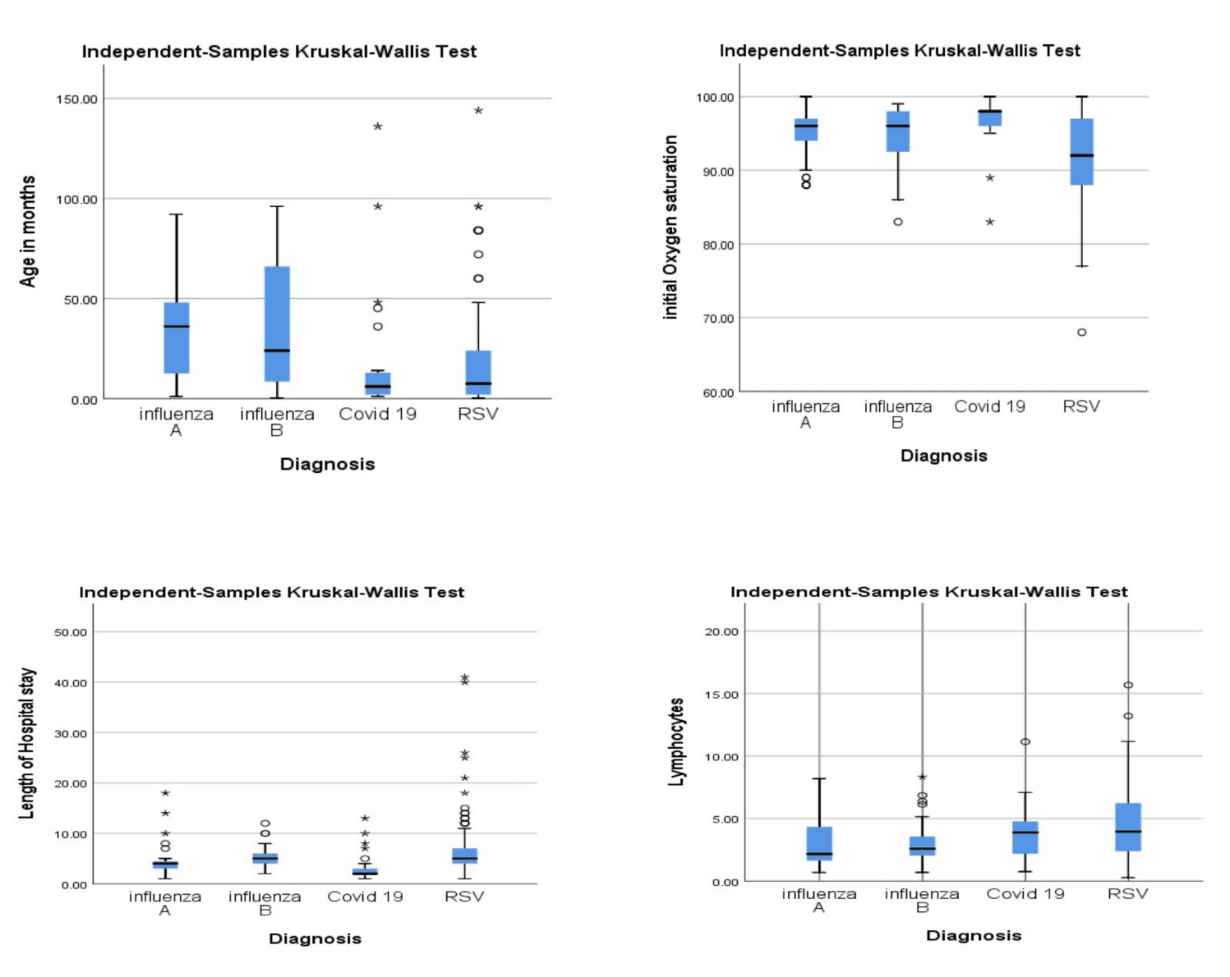
Correlation between viral infection and different parameters

A Kruskal-Wallis test was used to determine whether different viruses detected in children had an impact on their age, initial oxygen saturation, length of stay (LOS), and lymphocytic count (Figure 2). The results showed that there was a significant difference among the various viruses, indicating that the independent variable (virus type) had a notable effect on the dependent variables (age and initial oxygen saturation, LOS, and the lymphocytes). The p-value was less than 0.005, suggesting that these differences are statistically significant.

Table 4 indicates a significant clinical difference between COVID-19 and other viruses in terms of oxygen saturation, LOS, and total duration of illness. Notably, COVID-19 was associated with higher oxygen saturation, shorter LOS in the hospital, and a shorter total duration of illness compared to other viruses. These findings suggest that the clinical outcomes for COVID-19 were more favorable in these specific areas compared to other viral infections.

**Table 4:** Comparison between COVID-19 and non-COVID-19 viral infection

Parameter	COVID-19, N=25	Other viruses, N=212	P value
Initial oxygen saturation	98	95	<0.001
Length of hospital stay LOS	2	5	<0.001
Duration of the illness in days	5	9	<0.001
TLC	8.4	10.2	0.920
Lymphocytic count	3.9	3.5	0.658
Degree of fever on admission	38.1	37.8	0.220

Table 5 illustrates that children with COVID-19 had lower rates of PICU admission and required rescue medications less frequently than those with other respiratory viruses.

**Table 5:** Clinical variation between COVID-19 and non-COVID-19 infection

Variable	COVID-19, N=25	Non-COVID-19 viruses, N=212	P value
PICU admission	0	24%	0.051
Systemic steroid	16%	52.4%	0.001
Inhaled steroids	16%	49.5%	0.001
Inhaled bronchodilator	32%	71.7%	<0.001

## Discussion

Our study looked at the epidemiological and clinical data of children with bronchiolitis or flu who were admitted to our hospital after the COVID-19 pandemic season (from October 2022 to April 2023). We recruited 237 children. The peak number of cases occurred in November, accounting for 41% of the total recruited cases. RSV was identified as the predominant viral cause, responsible for 63.3% of the cases, which follows the known rate of infection worldwide, which is between 60 and 80% [1]. COVID-19 and influenza A and B cases were less frequent during this season, with rates of 10% and 13%, respectively.

A multicenter national study done in Italy desur study looked at the epidemiolocribed a marked increase in the absolute number of hospitalizations for viral bronchiolitis during the winter season of 2022-2023. In particular, the number of children who were hospitalized for viral bronchiolitis this winter was roughly double compared to the pre-pandemic seasons and presented a 70% increase compared to the 2021-2022 winter season. They reported a post-pandemic abrupt increase in bronchiolitis-related hospitalizations. The growing severity of the severity of the disease was evidenced by the requirement for more invasive respiratory support [13].

Abu-Raya et al. discussed some theories to explain this change, such as a decline in the children's immunity against RSV due to a decrease in viral exposure during the pandemic because of the lockdown and social distancing. Additionally, the interaction between the two viruses, which induced immune dysregulation, and finally the family's awareness and improvement of the health care system might play a role in the increasing number of cases [14].

Of the total patients, 21% required admission to the PICU, and most of them (37) received high-flow nasal cannula support, while eight required CPAP and six required intubations. These findings differed from what Wildenbeest et al. mentioned in 2023, which stated that infants with bronchiolitis often need hospitalization, with approximately 5% of them requiring ICU admission for respiratory monitoring and support [15]. This higher percentage can be explained in part by the increase in the number of cases in the post-COVID season and also by the naïve immune system of infants born during the lockdown period [13].

The determining factor for the outcome of bronchiolitis is the presence of co-morbidities such as prematurity and congenital heart disease (CHD) [3]. In our study, we had only 12 (5%) patients with CHD along with other co-morbidities who required prolonged hospital stay reaching 20 days. Only two patients needed PICU admission for high-flow nasal cannula (HFNC). Careful observation and good care helped them recover and land safely.

Despite this critical condition and PICU admission, all these children made a full recovery, and no deaths were reported. In agreement with Ghazaly et al., they found that most children had an uneventful course despite being hospitalized due to acute bronchiolitis [16].

Treatment options provided for those infants and children are supportive measures, in addition to medications such as inhaled bronchodilators and systemic and inhaled corticosteroids (67%, 48%, 46%), respectively, which indicates overuse of rescue medications in managing bronchiolitis. Despite all available guidelines that do not support or recommend routinely using these medications in bronchiolitis, it is still a common practice among physicians, which may be related to the short-term effect of corticosteroids [1].

The burden of RSV is high in the United States. It leads to hospitalization in 1-4% of infants and causes approximately 80,000 infant hospitalizations each year [17]. In the Kingdom of Saudi Arabia, in a recent study of children aged < 6 years of age (median five months) hospitalized with bronchiolitis, 53% and 64% of general ward admissions and PICU admissions, respectively, were due to RSV infection [18].

In addition to the high clinical burden, RSV infections are associated with a significant economic burden. Globally, it has been estimated that the total direct medical cost associated with acute medically attended lower respiratory tract disease (MA-LRTD) amounts to almost €5 billion, with just over half of the total cost spent on hospitalizations [19].

In our study, seasonal variations for each virus were different, showing distinct patterns across the year. RSV had a peak at the beginning of the season, but it gradually declined after that. However, what was known in the pre-COVID seasons is that RSV variability and its transmission are driven by geographical location and climate. In KSA, the circulation of the RSV is mainly confined to the winter season (from September to March), with a peak in December and January [20].

In contrast, influenza A and B maintained a relatively consistent presence throughout the season. Meanwhile, COVID-19 reached its peak during March and April. This is contrary to McClymont et al.'s publication in 2021, which highlighted that the climate variable might affect the viral transmission and infectivity in which variables including temperature and humidity can contribute to increased transmission of COVID-19, particularly in winter conditions through increased host susceptibility and viability of the virus. The available reports are inconclusive about this association as other variables such as humidity, rainfall, and wind speed exist. However, it is essential to know the seasonal variability of each country to implement the proper strategy for early warning systems and healthcare system preparation for the season [21].

Our study found that there was a statistically different age susceptibility between the different viruses, where the younger infants (median seven months) were hit mainly by RSV and COVID-19, followed by influenza A and B in the older ones. The age susceptibility of the different viral infections has been studied before and showed near results where enterovirus/rhinovirus and RSV constituted most of the viral respiratory infections among young children less than one year old. No coronavirus was detected among children between three and five years old. Co-infection caused by two or three respiratory viruses was detected in 52 patients (13.2%) [22].

Our study showed that respiratory viral infections have a significant effect on the initial oxygen saturation, where RSV is associated with more hypoxia than other viruses detected. Consequently, the LOS and the PICU admission rates were in favor of RSV, followed by influenza B, influenza A, and COVID-19, respectively. This was consistent with the finding of Fedorczak et al. in 2022, where they concluded that RSV infection in infants and young children had a more severe course than COVID-19 infection. They also found that RSV infection was associated with more extended hospitalization and that patients required more intensive support [23].

Lymphopenia is a consistent feature in viral infection, with different mechanisms that can vary between different viruses. Our study showed initial lymphocytic counts were significantly low in influenza B, influenza A, COVID-19, and RSV. Similar findings were noted in another study by Miron et al., where there was an increased lymphocyte count more common in infants with RSV, while lymphopenia was more frequent in infants with SARS-CoV-2 [24].

The lymphocytic count in children infected with influenza A and B showed the lowest recorded values across the different viral infections detected. Zhu et al.'s study concluded that a significantly lower lymphocyte count was observed in children with an influenza A or B infection than in those who are not infected, and no significant difference in the lymphocyte count was observed between children with influenza A and influenza B infections [25].

Lymphopenia is associated with disease severity. Seven different mechanisms were involved in lymphopenia caused by different viral infections, including cell death, elevated cytokines, chemokines and growth factors, inhibition of lymphopoiesis, lymphocyte trafficking, upregulated expression of co-inhibitory molecules, metabolic disorders, and elevated glucocorticoids [26].

The course of the COVID-19 pandemic was highly variable and associated with a significant community burden, morbidity, and mortality. Although published data suggested better outcomes in children compared to adults, there were concerns about the health of young children and their response to the virus after the lockdown ended, and they began attending schools and nurseries regularly. Our study showed that COVID-19, while significant, may lead to less severe outcomes in pediatric cases compared to other respiratory illnesses. Specifically, we found shorter hospital stays, less hypoxemia, and lower PICU admission rates compared to other studied respiratory viruses, as shown in Tables 4-5. Unlike the results of two US studies, hospitalization outcomes were found to be similar among those diagnosed with COVID-19 and those diagnosed with influenza [27,28].

Limitations of the study

The retrospective nature of the study and the small sample size are considered important limitations. Only four types of viruses were detected in the studied population, and their serotypes were unavailable, which hindered the identification of the co-infections and their correlation with the different clinical presentations.

## Conclusions

Our study showed that the prevalence of pediatric respiratory infections, mainly bronchiolitis, was higher following the pandemic and the release of lockdown measures; it was caused mainly by RSV, less frequently than influenza A, influenza B, and COVID-19. This resurgence was associated with more severe respiratory symptoms and an increased need for hospitalization. Notably, children with COVID-19 were in better condition compared to those with RSV. This difference can be attributed to the naïve immune systems of infants born during the lockdown and the decreased virulence of COVID-19 following the implementation of mass vaccination programs.

Bronchiolitis remains a significant burden on children and the healthcare system. Preventive strategies, including vaccination, should be implemented to mitigate this impact. Additionally, thorough preparation of the healthcare system is essential before the high season begins, ensuring adequate resources and support are in place to effectively manage the increased number of cases.
